# Developmental relations between internalizing symptoms and negative urgency during middle adolescence

**DOI:** 10.1017/S0954579426101357

**Published:** 2026-03-27

**Authors:** Jack T. Waddell, Natalia Cruz-Vespa, Fiona Baker, Duncan Clark, Bonnie Nagel, Kate B. Nooner, Susan F. Tapert, Wesley K. Thompson, Sandra A. Brown

**Affiliations:** 1 Psychology, Arizona State University, USA; 2 Psychiatry, UCSD, USA; 3 SRI International, USA; 4 Psychiatry, University of Pittsburgh, USA; 5 OHSU, USA; 6 Department of Psychology, University of North Carolina Wilmington, USA; 7 Population Neuroscience and Genetics Center, Laureate Institute for Brain Research, USA; 8 Psychology, University of California San Diego, USA

**Keywords:** Adolescence, impulsivity, internalizing symptoms, multilevel, negative urgency

## Abstract

Negative urgency is a transdiagnostic risk factor for a plethora of mental disorders. Internalizing symptoms are embedded in theories of negative urgency, yet we know little regarding how developmental changes in each coincide, and if changes in one predict changes in the other across middle adolescence. This study filled these voids in the literature, with *N* = 754 (52% female) community-recruited youth from the National Consortium on Alcohol NeuroDevelopment in Adolescence (NCANDA) study reporting internalizing symptoms and negative urgency annually. Negative urgency and internalizing symptoms were highly correlated at the between-person level, and between-person correlations were nearly double in size within male versus female adolescents. At the within-person level, changes in negative urgency and internalizing symptoms co-occurred across ages 14–18 but not age 13. Age 14 within-person changes in negative urgency prospectively predicted age 15 within-person changes in internalizing symptoms, and this effect was nearly double in size within female versus male adolescents. Findings held when accounting for externalizing symptoms, other impulsive personality traits, parenting, and school transitions. Results indicate that relations between negative urgency and internalizing symptoms were demonstrated across and within adolescents, with time-varying changes in negative urgency at age 14 being particularly impactful in terms of future internalizing symptoms.

## Introduction

In 2019, between 12% and 14% of adolescents age 13–18 reported criteria for at least one mental health disorder (Kieling et al., 2024). Mental health disorders have been on the rise since the early 2000s (Twenge et al., 2019), and recent research suggests an 8% increase in the number of emergency room visits associated with psychiatric/mental health disorders over the past decade (Theriault et al., 2020). Upticks in mental health struggles in adolescents have been noted to be a mental health crisis within the United States (CDC, 2024). Thus, it remains of high interest to developmental psychologists and prevention methodologists to better understand transdiagnostic risk factors for a broad range of mental health disorders to be targeted via prevention efforts.

One such risk factor is negative urgency, an impulsive personality trait defined as the predilection toward rash, ill-advised action and/or inaction when in a negative mood state (Cyders & Smith, 2008; Smith & Cyders, 2016). Negative urgency in adulthood is thought to map onto the higher-order personality traits of neuroticism (i.e., higher levels of distress and worry) and conscientiousness (i.e., thorough thoughtfulness and planning; Whiteside & Lynam, 2001). Developmental research conveys a similar story in adolescence, wherein the combination of high levels of childhood anger reactivity (i.e., frequent temper tantrums) and low effortful control (i.e., planful, thoughtful action) serves as a developmental predecessor to adolescent negative urgency (Waddell et al., 2021; Waddell et al., 2024). Nonetheless, research suggests that individuals who have higher levels of negative urgency are more likely to report symptoms associated with a host of mental health disorders and concerns, including substance use disorders (e.g., Coskunpinar et al., 2013; VanderVeen et al., 2016; Waddell et al., 2022), eating disorders (e.g., Davis et al., 2020; Fischer et al., 2012), affective disorders (e.g., Gunn et al., 2018; Pawluk & Koerner, 2016), personality disorders (e.g., Peters et al., 2017; Tragesser & Robinson, 2009), and suicidality (e.g., Anestis & Joiner, 2011; Picou et al., 2024). However, aside from early temperamental/personality correlates (e.g., Waddell et al., 2024; Whiteside & Lynam, 2001) and research into fluctuations in negative urgency across middle adolescence (Littlefield et al., 2016), we know little about the developmental etiology and course of negative urgency.

Central to theories of negative urgency is its links with internalizing symptoms (e.g., Cyders & Smith, 2008; Whiteside & Lynam, 2001). Theoretical models propose that individuals experiencing higher internalizing symptoms are more likely to develop negative urgency due to increased vulnerability to dysregulated affect (Berg et al., 2015). For example, individuals with elevated levels of negative urgency are more likely to use disengagement or reflexive emotion regulation strategies, which are each associated with internalizing symptoms (Aldao et al., 2010; King et al., 2018). Further, studies have shown that negative urgency acts as a mechanism through which internalizing symptoms relate to a variety of mental health concerns, implying directionally from internalizing symptoms to negative urgency (Felton et al., 2020; Guillot et al., 2014; Pang et al., 2014). However, longitudinal studies have also shown the opposite, that negative urgency is associated with increased internalizing symptoms (Riley & Smith, 2017; Smith et al., 2013; Woods-Gonzalez et al., 2024). Thus, the directionality and temporal precedence of links between negative urgency and internalizing symptoms remain unclear. It also remains unclear how such directional relations exist within male/female youth, given that female youth often exhibit higher levels of internalizing symptoms (e.g., Bennett et al., 2005; Khesht-Masjedi et al., 2017) and negative urgency (e.g., Littlefield et al., 2016; Santano-Mogena et al., 2023).

Furthermore, we know little regarding *when* relations between internalizing symptoms and negative urgency may manifest across adolescent development. Theory has viewed personality as relatively static for decades (e.g., Caspi et al., 2005; McCrae & Costa, 1997), however modern research suggests substantial shifts and fluctuations in personality traits, particularly impulsivity, during middle adolescence (e.g., Waddell & Sasser, 2024; Wasserman et al., 2020). Therefore, it is possible that the two have time-varying dynamic relations that occur at specific times during adolescent development, when fluctuations in impulsive personality traits are substantial. Research suggests that stress exposure is associated with increases and then decreases in impulsivity from early to middle-adolescence (Wasserman et al., 2023). However, research to date has yet to test such age-varying relations specific to negative urgency, as well as the reciprocal time-varying influences of internalizing symptoms and negative urgency on one another to truly disentangle temporal precedence. If time-varying relations are found, personalized early interventions would gain knowledge regarding the developmental timing and temporal relations between the two.

Given that time-varying associations between negative urgency and internalizing symptoms have yet to be directly tested, little is known about the robustness of these relations above and beyond other theoretically relevant factors. Internalizing symptoms are strongly correlated with externalizing symptoms (e.g., Achenbach et al., 2016; Curci et al., 2023), and thus it is unclear whether potential effects are specific to internalizing processes or reflect broader psychopathology. Similarly, negative urgency is highly correlated with other impulsive traits (e.g., Cyders et al., 2014; Waddell et al., 2022), and thus it is equally unclear whether associations with internalizing symptoms are unique to negative urgency or instead attributable to impulsivity more broadly (e.g., Cyders et al., 2014; Watts et al., 2020). In addition, parenting behavior has been shown to predispose youth to both heightened negative urgency and elevated internalizing symptoms (e.g., Dallaire et al., 2006; Waddell et al., 2024). Thus, parenting practices may partially account for any observed association between these constructs. Finally, developmental transitions into and out of schooling may also shape these relations, as such transitions are associated with increases in negative affectivity (e.g., Fraser et al., 2021) and impulsive traits, including negative urgency (LaSpada et al., 2020; Littlefield et al., 2016). Taken together, clarifying whether time-varying associations between negative urgency and internalizing symptoms persist after accounting for these potential confounds is critical in refining developmental models of how both constructs emerge and are maintained across middle adolescence.

Therefore, the current study used data from a large, longitudinal study of adolescent development to understand the developmental links between internalizing symptoms and negative urgency, both across and within individuals. The current study used random intercept cross-lagged panel models (RI-CLPMs) to test relations between internalizing symptoms and negative urgency across ages 13–18. It was hypothesized that, across individuals, the random intercepts of negative urgency and internalizing symptoms would be correlated, such that higher levels of negative urgency would be associated with higher levels of internalizing symptoms at the between-person level. It was also hypothesized that age-specific within-person changes in levels of negative urgency and internalizing symptoms would be contemporaneously correlated, showing correlated change at a given age. Further, it was hypothesized that within-person changes in levels of negative urgency at a given age (e.g., age 13) would predict within-person changes one year later in levels of internalizing symptoms (e.g., age 14), and vice versa. Such reciprocal relations were hypothesized across age, however we anticipated effects to be largest during age 13–14, given prior research showing stress and impulsive personality traits are positively associated during this age period (Wasserman et al., 2023). Models were also estimated within male and female youth separately, however these analyses were considered exploratory.

## Methods

### Participants and procedure

Participants (*N* = 754) came from the community-based cohort of the National Consortium on Alcohol and Neurodevelopment in Adolescence to Adulthood (NCANDA-A) Study, a multi-site longitudinal study investigating the impact of substance use on neurodevelopmental trajectories. A total of 831 individuals ages 12 to 21 were recruited from five sites across the United States, including University of Pittsburgh, SRI International, Duke University, Oregon Health & Science University, and UC San Diego to complete annual visits comprised of behavioral and clinical assessments, neurocognitive tests, and functional MRI scans. Participants in the present study gave assent if less than 18 years old and consent once they reached 18 years of age to trained research assistants. Each site obtained IRB approval; minors provided written assent with parental consent, and adults provided written informed consent at each annual visit. The majority of participants (83%) had no or limited previous exposure to alcohol and other substances at baseline, while the rest (17%) exceeded national age averages of substance use frequency. Baseline exclusion criteria for participation included (1) current diagnosis of severe Axis I psychiatric disorders (e.g., schizophrenia, bipolar disorder), (2) current use of psychoactive medication, (3) serious medical problems prohibiting protocol completion, (4) lack of parental involvement, (5) limited English fluency, (6) known prenatal drug or alcohol exposure, or (7) any MRI contradictions that could interfere with neuroimaging (see Brown et al., 2015 for more details).

Data from NCANDA Data Release 9.0 were used for the current study’s analyses (NCANDA_RELEASE_9Y_REDCAP_MEASUREMENTS_V01), which included data from a baseline interview up to the nine-year follow-up visit. Participants with data during middle adolescence, defined as age 13–18 (e.g., Sawyer et al., 2018; Steinberg, 2014), were retained in the current study, totaling *N* = 754 participants (see Table 1 for descriptive statistics and sample demographics). Due to the sequential cohort design, *N* = 189 cases from age 13, *N* = 265 cases from age 14, *N* = 365 cases from age 15, *N* = 424 cases from age 16, *N* = 474 cases from age 17, and *N* = 485 cases from age 18 were included in analyses.

**Table 1.** Descriptive statistics

	Mean or %	SD
**Demographic variables**		
Sex Male Female	50% 50%	
Race/Ethnicity White Black/African American Asian American Indian/Alaskan Native Native Hawaiian/Pacific Islander Other	71.3% 12.2% 7.1% 0.4% 0.5% 8.5%	
**Means across time**		
Age 13 Negative Urgency	1.95	0.57
Age 14 Negative Urgency	1.95	0.69
Age 15 Negative Urgency	1.97	0.75
Age 16 Negative Urgency	1.93	0.70
Age 17 Negative Urgency	1.91	0.69
Age 18 Negative Urgency	1.87	0.67
**Means across time**		
Age 13 Internalizing Symptoms	51.7	3.77
Age 14 Internalizing Symptoms	52.5	4.48
Age 15 Internalizing Symptoms	53.0	5.94
Age 16 Internalizing Symptoms	53.3	5.36
Age 17 Internalizing Symptoms	53.4	5.73
Age 18 Internalizing Symptoms	53.0	5.29

*Note.* Participants (*N* = 754) come from the National Consortium on Alcohol and Neurodevelopment in Adolescence to Adulthood (NCANDA-A), a multi-site longitudinal study investigating the impact of substance use on neurodevelopmental trajectories; Negative urgency was measured on a scale of 1 (Agree Strongly) to 4 (Disagree Strongly) and reverse scored such that higher scores were indicative of higher levels of negative urgency; Internalizing symptoms were scaled T-scores with a score of 50 set as the mean and 10 points being 1 standard deviation form the mean.

### Measures

### Primary measures

#### Internalizing symptoms

Internalizing symptoms were assessed annually via the Diagnostic and Statistical Manual 5 (DSM-5) scales of the Youth Self-Report (YSR; Achenbach & Rescorla, 2001) and Adult Self Report (ASR; Achenbach & Rescorla, 2003). The internalizing symptoms scale from the YSR/ASR, made up of items from the anxious/depressed (13 items on YSR; 18 items on ASR), withdrawn/depressed (8 items on YSR; 9 items on ASR) and somatic complaints (10 items on YSR; 12 items on ASR) subscales. Each symptom was rated on a scale of 0 = not true, 1 = somewhat true, to 2 = very true. A scaled t-score (*a* = .74) for the internalizing scale was computed for each year of assessment and extracted for analyses.

#### Impulsive personality traits

The 20-item Brief Urgency Premeditation Perseverance Sensation Seeking (B-UPPS; Cyders et al., 2014) Scale was used to assess impulsive personality traits. Negative urgency (e.g., “When I am upset, I often act without thinking”), positive urgency (e.g., “When I am very happy, I can’t seem to stop myself from doing things that can have bad consequences”), lack of premeditation (“I like to stop and think things over before I do them” [recoded]), lack of perseverance (e.g., “Unfinished tasks really bother me” [recoded]), and sensation seeking (e.g., “I generally seek new and exciting experiences and sensations”) were assessed on a scale of 1 (Agree Strongly) to 4 (Disagree Strongly). Items were recoded such that higher values were indicative of higher levels of each impulsive personality trait. All subscales showed good reliability (*a* = .68–.82).

#### Secondary measures

##### Externalizing symptoms

Externalizing symptoms were assessed annually via the Diagnostic and Statistical Manual 5 (DSM-5) scales of the Youth Self-Report (YSR; Achenbach & Rescorla, 2001) and Adult Self Report (ASR; Achenbach & Rescorla, 2003). The externalizing symptoms scale from the YSR/ASR, made up of items from the rule breaking behavior (15 items on YSR; 14 items on ASR) and aggressive behavior (17 items on YSR; 15 items on ASR) subscales. Each symptom was rated on a scale of 0 = not true, 1 = somewhat true, to 2 = very true. A scaled t-score (*a* = .64) for the externalizing scale was computed for each year of assessment and extracted for analyses.

##### Parenting

Parental support and parental monitoring were measured via adapted items from previous research by Stattin & Kerr (2000). Six items measured parental support (e.g., “I can count on my father/mother to help me out if I have some kind of a problem”) reported on a scale of 1 (never) to 4 (always). Five items measured parental monitoring by asking participants to report the extent to which parents know about several aspects of their lives (e.g., what you do with your free time, who your friends are) on a scale of 1 (don’t really know) to 3 (always really know). Average scores for each, which had adequate reliability (*a* = .81–.85), were extracted for analyses.

##### School transitions

Participants reported if they were in middle school (1), high school (2), or college (3) across all waves of data collection. When participants reported a change in schooling (i.e., middle school to high school; high school to college), this transition was marked as a 1 (1 = school transition vs. 0 = no school transition).

##### Sex

Participants reported their biological sex during the baseline interview (0 = female, 1 = male).

##### Socioeconomic status

Socioeconomic status was measured as the years of combined parental education, in line with prior studies from the NCANDA consortium (e.g., Patel et al., 2024; Sullivan et al., 2017).

### Data analytic plan

#### Primary analyses

The current study estimated random-intercept cross-lagged panel models (RI-CLPM; Hamaker et al., 2015) to test study hypotheses. Importantly, the RI-CLPM is robust compared to standard cross-lagged panel models in that it disaggregates within-person change from between-person variation that remains static across time, allowing a more parsimonious test of reciprocal influences (e.g., Hamaker et al., 2015; Usami et al., 2019). Thus, there were four parameters of the RI-CLPM of interest: (1) random intercepts over time, which assess between-person, “trait-like” levels of each outcome across time, (2) within-person correlations, which assess the contemporaneous covariance between within-person changes in two outcomes at a given age, (3) autoregressive pathways, which assess the “carryover” of within-person change affecting subsequent within-person change in a given outcome at a given age, and (4) reciprocal pathways, which assess the impact of within-person changes in one variable at a given age on subsequent within-person changes in the same variable a year later (Hamaker et al., 2015).

For the current study, a RI-CLPM was estimated to test the developmental relations between internalizing symptoms and negative urgency across ages 13–18. Thus, random intercepts for internalizing symptoms and negative urgency across ages 13–18 were specified and allowed to correlate with one another. Further, within-person residual variability in internalizing symptoms and negative urgency at each age were converted into a phantom variable, which were correlated at each age, testing within-person correlated change across ages 13–18. Phantom variables were created such that residual within-person variability was specified and thus allowed for prediction of (and from) such variability. Therefore, each phantom variable was then specified as a predictor of the subsequent phantom one year later, testing within-person autoregressive effects, as well as the phantom of the other variable a year later, testing within-person reciprocal effects. At the between-person level, sex at birth and socioeconomic status were specified as predictors of the random intercepts of internalizing symptoms and negative urgency – sex and socioeconomic status were allowed to freely covary.

We first specified the model in the full sample, followed by a model estimated separately within male youth and within female youth to assess within-group differences by sex at birth. Given research demonstrating stronger within-sex rather than between-sex differences (e.g., Hyde, 2005), we sought to compare within-group differences in female youth and male youth, rather than comparing the two against one another. Furthermore, given that one of the limitations of RI-CLPM models is its already-high number of parameters and model constraints necessary (e.g., Hamaker et al., 2015), we were leery to additional parameterization and constraints to compare across groups. Adequate model fit was defined as having RMSEA values near.06, CFI and TLI values near .95, and SRMR values near .08 (Hu & Bentler, 1999). All analyses were conducted in MPlus Version 8.9 using Maximum Likelihood Estimation with Robust Standard Errors (MLR), and missing data was estimated via Full Information Maximum Likelihood (FIML). All model parameters are reported as standardized effects.

#### Sensitivity analyses

A series of sensitivity analyses were estimated to ensure robustness of the model. First, sensitivity analyses were estimated with externalizing symptoms in the model alongside internalizing symptoms, allowing understanding of unique aspects of relations between internalizing versus externalizing symptoms and negative urgency. Models were built identically to above, with the addition of a random intercept for externalizing symptoms and the inclusion of externalizing symptoms as phantom variables in the within-person part of the model. Second, sensitivity analyses were estimated with other impulsive personality traits in the model (i.e., positive urgency, lack of premeditation, lack of perseverance, sensation seeking) to test whether relations with internalizing symptoms were specific to negative urgency or were present across a swath of impulsive personality traits. Models were estimated identically to the primary model, with the addition of random intercepts and phantom variables for the other four impulsive personality traits. Third, sensitivity analyses were estimated in a sample of individuals (*N* = 351) who reported clinically elevated internalizing symptoms during one of the time points across the study period. Similar to within-sex models, we sought to model how the theoretical model of interest operated within the group of individuals with clinically significant internalizing symptoms, rather than if certain parameters differed from the group of individuals who did not report clinically significant symptoms.

##### Exploratory analyses

Exploratory analyses incorporated parenting variables into the model, namely parental monitoring and parental warmth, to test whether inclusion of parenting affected any study fundings. Such sensitivity analyses were first estimated with parental monitoring as a random intercept/phantom variable and then with parental warmth as a random intercept/phantom variable. Finally, sensitivity analyses tested whether model parameters were changed when accounting for school transitions at a given time point, defined as whether an adolescent transitioned from middle school to high school or from high school to college. Of note, transitions were only present at ages 13, 14, and 17, and thus no schooling variables were entered into the model at ages 15 and 16.

## Results

Model fit indices across all models are displayed in Table 2.

**Table 2.** Model fit indices across primary and sensitivity models

	*X* ^2^ (*p*-value)	RMSEA	CFI	TLI	SRMR
**Primary analyses:**					
Full Sample	106.69 (*p* < .001)	.033	.971	.955	.063
Male Youth	72.91 (*p* < .009)	.040	.970	.951	.071
Female Youth	72.02 (*p* < .011)	.040	.969	.949	.070
**Sensitivity analyses:**					
Clinical Levels Model	66.70 (*p* < .08)	.027	.976	.959	.057
Other UPPS-P Model	615.6 (*p* < .001)	.027	.969	.945	.062
Externalizing Model	197.73 (*p* < .001)	.031	.976	.960	.061
**Exploratory analyses:**					
Parental Monitoring Model	156.47 (*p* < .006)	.022	.982	.970	.051
Parental Support Model	159.97 (*p* < .001)	.025	.979	.966	.057
School Transitions Model	163.86 (*p* < .001)	.030	.962	.950	.073

*Note.* Adequate model fit was defined as RMSEA values near or under .06, CFI/TLI values near or above .95, and SRMR values near or below .08.

### Primary model

The full sample random-intercept cross-lagged panel model fit the data well, and all factor loadings onto the random intercepts of negative urgency and internalizing were statistically significant, showing substantial between-person variation in each outcome. Further, the random intercepts of negative urgency and internalizing symptoms were highly correlated, suggesting that individuals with higher levels of internalizing symptoms also reported higher levels of negative urgency across ages 13–18 (see Table 3 for model parameters). Higher socioeconomic status was associated with lower values on the random intercept of negative urgency and the random intercept of internalizing symptoms. Sex was unrelated to the random intercept of negative urgency but male sex was related to lower values on the random intercept of internalizing symptoms.

**Table 3.** Primary RI-CLPM parameters

	*b*	SE	*p*-value
**Random intercepts**			
Negative urgency – Internalizing	.50	.07	< .001
**Predictors of random intercepts**			
Negative urgency			
Socioeconomic status	−.15	.05	.004
Sex	.03	.05	.50
Internalizing symptoms			
Socioeconomic status	−.11	.05	.041
Sex	−.13	.05	.009
**Correlated change**			
Age 13 Negative Urgency – Internalizing	.13	.12	.27
Age 14 Negative Urgency – Internalizing	.28	.09	.003
Age 15 Negative Urgency – Internalizing	.23	.08	.003
Age 16 Negative Urgency – Internalizing	.32	.05	< .001
Age 17 Negative Urgency – Internalizing	.25	.06	< .001
Age 18 Negative Urgency – Internalizing	.32	.06	< .001
**Autoregressive pathways**			
Age 13 Negative Urgency → Age 14 Negative Urgency	.01	.13	.98
Age 14 Negative Urgency → Age 15 Negative Urgency	.23	.10	.014
Age 15 Negative Urgency → Age 16 Negative Urgency	.33	.08	< .001
Age 16 Negative Urgency → Age 17 Negative Urgency	.23	.08	.003
Age 17 Negative Urgency → Age 18 Negative Urgency	.37	.07	< .001
Age 13 Internalizing → Age 14 Internalizing	.23	.12	.049
Age 14 Internalizing → Age 15 Internalizing	.23	.10	.019
Age 15 Internalizing → Age 16 Internalizing	.36	.10	< .001
Age 16 Internalizing → Age 17 Internalizing	.49	.07	< .001
Age 17 Internalizing → Age 18 Internalizing	.46	.08	< .001
**Prospective pathways**			
Age 13 Negative Urgency → Age 14 Internalizing	−.09	.12	.49
Age 14 Negative Urgency → Age 15 Internalizing	.13	.07	.045
Age 15 Negative Urgency → Age 16 Internalizing	−.04	.08	.64
Age 16 Negative Urgency → Age 17 Internalizing	.06	.07	.46
Age 17 Negative Urgency → Age 18 Internalizing	.02	.06	.76
Age 13 Internalizing → Age 14 Negative Urgency	.16	.12	.20
Age 14 Internalizing → Age 15 Negative Urgency	−.05	.09	.57
Age 15 Internalizing → Age 16 Negative Urgency	.10	.08	.22
Age 16 Internalizing → Age 17 Negative Urgency	.07	.07	.33
Age 17 Internalizing → Age 18 Negative Urgency	.10	.07	.17.

*Note.* RI-CLPM stands for random intercept cross-lagged panel model.

**Figure 1.**
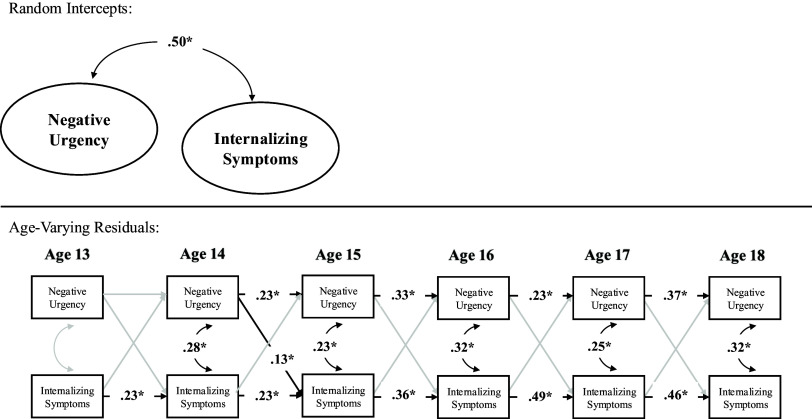
Random intercept cross-lagged panel model across full sample. *Note.* *p < .05.

In terms of contemporaneous change, within-person deviations (variations) in internalizing symptoms were correlated with within-person deviations in negative urgency at ages 14–18 but not at age 13. Thus, across ages 14–18, a change in internalizing symptoms was correlated with a contemporaneous change in negative urgency. Prospectively, within-person deviations in internalizing symptoms did not significantly predict subsequent deviations in negative urgency across ages. By contrast, within-person deviations in negative urgency, however, predicted subsequent deviations in internalizing symptoms from age 14–15 but not from ages 13–14 nor ages 15–16, 16–17, nor 17–18. Further, the autoregressive stability path from within-person deviations in negative urgency to subsequent deviations in negative urgency were statistically significant across ages 14–18 but not ages 13–14, and the stability path from within-person deviations in internalizing symptoms to subsequent deviations in internalizing symptoms were statistically significant from ages 13–18 (see Table 3 and Figure 1).

### Male youth model

The random-intercept cross-lagged panel model within male youth fit the data well. All factor loadings onto the random intercepts of negative urgency and internalizing were statistically significant, showing substantial between-person variation in each variable. Further, the random intercepts of negative urgency and internalizing symptoms were highly correlated, suggesting that male youth with higher levels of internalizing symptoms also reported higher levels of negative urgency across ages 13–18 (see Table 4 for model parameters). Higher socioeconomic status was associated with lower scores on the random intercept of negative urgency and the random intercept of internalizing symptoms.

**Table 4.** Male youth RI-CLPM parameters

	*b*	SE	*p*-value
**Random intercepts**			
Negative Urgency – Internalizing	.56	.11	< .001
**Predictors of random intercepts**			
Negative urgency			
Socioeconomic status	−.16	.08	.043
Internalizing symptoms			
Socioeconomic status	−.18	.07	.01
**Correlated change**			
Age 13 Negative Urgency – Internalizing	.29	.16	.073
Age 14 Negative Urgency – Internalizing	.36	.12	.003
Age 15 negative Urgency – Internalizing	.22	.10	.027
Age 16 Negative Urgency – Internalizing	.34	.08	< .001
Age 17 Negative Urgency – Internalizing	.27	.07	< .001
Age 18 Negative Urgency – Internalizing	.31	.10	.001
**Autoregressive pathways**			
Age 13 Negative Urgency → Age 14 Negative Urgency	−.01	.22	.98
Age 14 Negative Urgency → Age 15 Negative Urgency	.19	.15	.19
Age 15 Negative Urgency → Age 16 Negative Urgency	.27	.10	.009
Age 16 Negative Urgency → Age 17 Negative Urgency	.30	.11	.007
Age 17 Negative Urgency → Age 18 Negative Urgency	.40	.08	< .001
Age 13 Internalizing → Age 14 Internalizing	.27	.16	.085
Age 14 Internalizing → Age 15 Internalizing	.32	.12	.006
Age 15 Internalizing → Age 16 Internalizing	.36	.13.	.008
Age 16 Internalizing → Age 17 Internalizing	.58	.10	< .001
Age 17 Internalizing → Age 18 Internalizing	.50	.10	< .001
**Prospective pathways**			
Age 13 Negative Urgency → Age 14 Internalizing	−.10	.20	.61
Age 14 Negative Urgency → Age 15 Internalizing	.05	.09	.55
Age 15 Negative Urgency → Age 16 Internalizing	−.02	.10	.81
Age 16 Negative Urgency → Age 17 Internalizing	−.01	.09	.89
Age 17 Negative Urgency → Age 18 Internalizing	.07	.08	.42
Age 13 Internalizing → Age 14 Negative Urgency	−.10	.20	.61
Age 14 Internalizing → Age 15 Negative Urgency	−.07	.14	.59
Age 15 Internalizing → Age 16 Negative Urgency	.10	.12	.40
Age 16 Internalizing → Age 17 Negative Urgency	.13	.10	.18
Age 17 Internalizing → Age 18 Negative Urgency	.10	.09	.31

*Note.* RI-CLPM stands for random intercept cross-lagged panel model.

**Figure 2.**
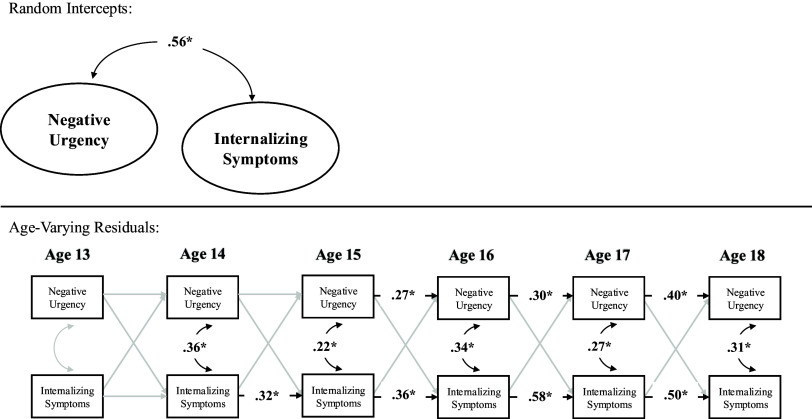
Random intercept cross-lagged panel model within male youth. *Note.* *p < .05.

In terms of correlated change, within-person deviations in internalizing symptoms were correlated with within-person deviations in negative urgency at ages 14–18 but not at age 13. Thus, across ages 14–18, a change in internalizing symptoms in male youth was associated with a correlated change in negative urgency. Prospectively, within-person deviations in internalizing symptoms did not predict subsequent deviations in negative urgency across ages. Similarly, within-person deviations in negative urgency did not predict subsequent deviations in internalizing across ages. Further, the stability path from within-person deviations in negative urgency to subsequent deviations in negative urgency were statistically significant across ages 15–18 but not ages 13–14 nor ages 14–15. Similarly, the stability path from within-person deviations in internalizing symptoms to subsequent deviations in internalizing symptoms were statistically significant from age 14–18 but not from age 13–14 (see Table 4 and Figure 2).

### Female youth model

The random-intercept cross-lagged panel model within female youth fit the data well. All factor loadings onto the random intercepts of negative urgency and internalizing were statistically significant, showing substantial between-person variation in each variable. Further, the random intercepts of negative urgency and internalizing symptoms were moderately correlated, suggesting that female youth with higher levels of internalizing symptoms also reported higher levels of negative urgency across ages 13–18 (see Table 5). Higher socioeconomic status was unrelated to values on the random intercept of negative urgency and internalizing symptoms.

**Table 5.** Female Youth RI-CLPM Parameters

	*b*	SE	*p*-value
**Random Intercepts**			
Negative Urgency – Internalizing	.40	.11	< .001
**Predictors of Random Intercepts**			
Negative Urgency			
Socioeconomic Status	−.05	.07	.45
Internalizing Symptoms			
Socioeconomic Status	.13	08	.091
**Correlated Change**			
Age 13 Negative Urgency – Internalizing	−.10	.16	.55
Age 14 Negative Urgency – Internalizing	.22	.16	.17
Age 15 Negative Urgency – Internalizing	.26	.14	.064
Age 16 Negative Urgency – Internalizing	.30	.07	< .001
Age 17 Negative Urgency – Internalizing	.29	.09	.001
Age 18 Negative Urgency – Internalizing	.34	.08	< .001
**Autoregressive pathways**			
Age 13 Negative Urgency → Age 14 Negative Urgency	−.01	.19	.94
Age 14 Negative Urgency → Age 15 Negative Urgency	.24	.14	.077
Age 15 Negative Urgency → Age 16 Negative Urgency	.43	.10	< .001
Age 16 Negative Urgency → Age 17 Negative Urgency	.17	.12	.13
Age 17 Negative Urgency → Age 18 Negative Urgency	.30	.12	.013
Age 13 Internalizing → Age 14 Internalizing	.23	.18	.20
Age 14 Internalizing → Age 15 Internalizing	.10	.17	.56
Age 15 Internalizing → Age 16 Internalizing	.37	.14	.008
Age 16 Internalizing → Age 17 Internalizing	.40	.11	< .001
Age 17 Internalizing → Age 18 Internalizing	.42.	11	< .001
**Prospective pathways**			
Age 13 Negative Urgency → Age 14 Internalizing	−.16	.19	.41
Age 14 Negative Urgency → Age 15 Internalizing	.27	.13	.032
Age 15 Negative Urgency → Age 16 Internalizing	−.02	.13	.90
Age 16 Negative Urgency → Age 17 Internalizing	.13	.11	.25
Age 17 Negative Urgency → Age 18 Internalizing	−.01	.10	.89
Age 13 Internalizing → Age 14 Negative Urgency	.34	.13	.009
Age 14 Internalizing → Age 15 Negative Urgency	−.02	.13	.86
Age 15 Internalizing → Age 16 Negative Urgency	.12	.11	.27
Age 16 Internalizing → Age 17 Negative Urgency	.07	.13	.58
Age 17 Internalizing → Age 18 Negative Urgency	.13	.12	.29

*Note.* RI-CLPM stands for random intercept cross-lagged panel model.

**Figure 3.**
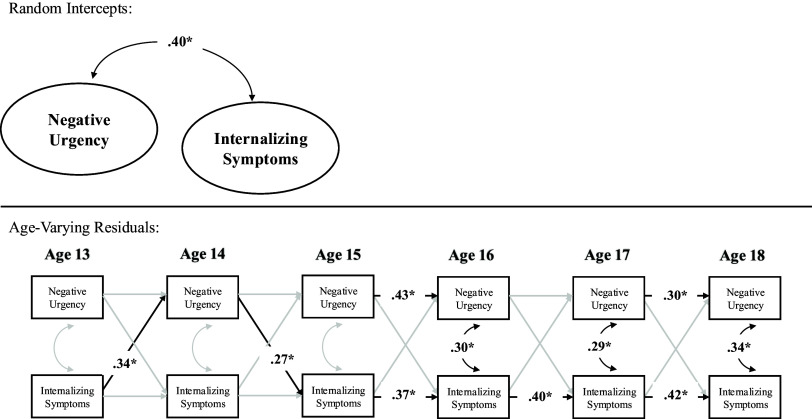
Random intercept cross-lagged panel model within female youth. *Note.* *p < .05.

In terms of correlated change, within-person deviations in internalizing symptoms were correlated with within-person deviations in negative urgency at age 16–18 but not age 13–15. Thus, across ages 16–18, a change in internalizing symptoms was associated with a contemporaneous change in negative urgency. Prospectively, within-person deviations in internalizing symptoms at age 13 predicted within-person deviations in negative urgency at age 14, and within-person deviations in negative urgency at age 14 predicted within-person deviations in internalizing symptoms at age 15. However, within-person deviations in internalizing symptoms did not predict subsequent deviations in negative urgency across ages 14–18, nor did within-person deviations in negative urgency predict subsequent deviations in internalizing across ages 13–14 nor 15–18. Further, the stability path from within-person deviations in negative urgency to subsequent deviations in negative urgency were statistically significant across ages 15–16 and ages 17–18 but not ages 13–15 nor 16–17. Similarly, the stability path from within-person deviations in internalizing symptoms to subsequent deviations in internalizing symptoms were statistically significant from ages 15–18 but not ages 13–15 (see Table 5 and Figure 3).

### Sensitivity analyses

**Figure 4.**
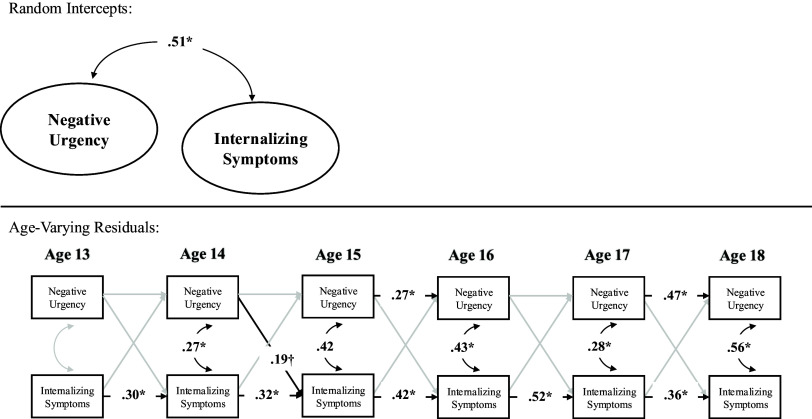
Random intercept cross-lagged panel model within those reporting clinical levels of internalizing symptoms. Note. This model was estimated in *N* = 356 youth who reported clinically elevated internalizing symptoms at one point throughout age 13-18; findings were largely the same as the primary model, albeit the pathway from age 14 negative urgency to age 15 internalizing symptoms increased in effect size but had more error and was not statistically significant. *p < .05.

**Clinical levels of internalizing.** Sensitivity analyses within a subsample of participants that reported clinically elevated internalizing symptoms (*N* = 351), which can be seen in Figure 4 and Table 6, fit the data well. The random intercepts of negative urgency and internalizing symptoms remained moderately correlated, suggesting that, in a subsample of youth who report elevated internalizing symptoms, higher levels of internalizing symptoms were associated with higher levels of negative urgency across ages 13–18 (see Supplemental Table 1). In terms of correlated change, within-person deviations in internalizing symptoms were correlated with within-person deviations in negative urgency between ages 14–18 but not ages 13–14. Thus, across ages 14–18, a change in internalizing symptoms was associated with a contemporaneous change in negative urgency. In terms of prospective pathways, the effect size of within-person increases in negative urgency at age 14 on within-person increases in internalizing symptoms at age 15 was larger in magnitude yet did not meet threshold for statistical significance than the primary model that had more than double the sample size (*b* = .19, SE = .10, *p* = .056). There were no additional statistically significant within-person prospective relations between internalizing symptoms and negative urgency.

**Table 6.** RI-CLPM Parameters For Those Reporting Clinical Level Internalizing Symptoms

	*b*	SE	*p*-value
**Random Intercepts**			
Negative Urgency – Internalizing	.51	.13	< .001
**Predictors of random intercepts**			
Negative Urgency			
Socioeconomic Status	−.10	09	.26
Sex	.01	.08	.98
Internalizing Symptoms			
Socioeconomic Status	−.11	.09	.23
Sex	−.21	.10	.04
**Correlated change**			
Age 13 Negative Urgency – Internalizing	.21	.15	.15
Age 14 Negative Urgency – Internalizing	.27	.14	.048
Age 15 Negative Urgency – Internalizing	.42	.11	< .001
Age 16 Negative Urgency – Internalizing	.43	.07	< .001
Age 17 Negative Urgency – Internalizing	.28	.09	.002
Age 18 Negative Urgency – Internalizing	.56	.09	< .001
**Autoregressive pathways**			
Age 13 Negative Urgency → Age 14 Negative Urgency	.18	.17	.31
Age 14 Negative Urgency → Age 15 Negative Urgency	.15	.15	.31
Age 15 Negative Urgency → Age 16 Negative Urgency	.27	.12	.025
Age 16 Negative Urgency → Age 17 Negative Urgency	.20	.12	.098
Age 17 Negative Urgency → Age 18 Negative Urgency	.47	.12	< .001
Age 13 Internalizing → Age 14 Internalizing	.30	.15	.041
Age 14 Internalizing → Age 15 Internalizing	.32	.12	.009
Age 15 Internalizing → Age 16 Internalizing	.42	.12	< .001
Age 16 Internalizing → Age 17 Internalizing	.52	.09	< .001
Age 17 Internalizing → Age 18 Internalizing	.36	.13	.006
**Prospective pathways**			
Age 13 Negative Urgency → Age 14 Internalizing	−.03	.16	.84
Age 14 Negative Urgency → Age 15 Internalizing	.19	.10	.056
Age 15 Negative Urgency → Age 16 Internalizing	−.01	.12	.92
Age 16 Negative Urgency → Age 17 Internalizing	.13	.12	.26
Age 17 Negative Urgency → Age 18 Internalizing	.11	.12	.34
Age 13 Internalizing → Age 14 Negative Urgency	.12	.18	.49
Age 14 Internalizing → Age 15 Negative Urgency	.23	.12	.061
Age 15 Internalizing → Age 16 Negative Urgency	.11	.12	.37
Age 16 Internalizing → Age 17 Negative Urgency	.13	.11	24
Age 17 Internalizing → Age 18 Negative Urgency	−.17	.16	.28

*Note.* RI-CLPM stands for random intercept cross-lagged panel model.

**Figure 5.**
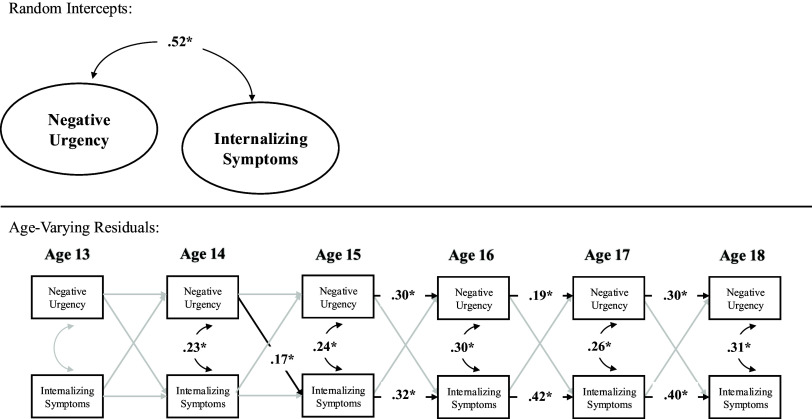
Random intercept cross-lagged panel model covarying other impulsive personality traits. Note. This model controlled for the random intercepts of positive urgency, lack of premeditation, lack of perseverance, and sensation seeking, as well as contemporaneous correlated change and prospective relations with internalizing. However, as seen above, primary paths between negative urgency and internalizing were unchanged. *p < .05.

**Other impulsive personality traits.** Sensitivity analyses incorporating other impulsive personality traits, which can be seen in Figure 5, fit the data well. Above and beyond random intercept correlations between internalizing symptoms and positive urgency (*b* = −.45, SE = .08, *p* < .001), lack of premeditation (*b* = .26, SE = .10, *p* = .007), lack of perseverance (*b* = .20, SE = .11, *p* = .074), and sensation seeking (*b* = −.01, SE = .08, *p* = .88), the random intercepts of internalizing symptoms and negative urgency remained statistically significant (see Supplemental Table 2). Correlated change between internalizing symptoms and negative urgency remained across ages 14–18 but not age 13, even when accounting for contemporaneous correlated change between internalizing symptoms and other impulsive personality traits. In terms of prospective pathways, the effect size of within-person increases in negative urgency at age 14 on within-person increases in internalizing symptoms at age 15 was statistically significant and slightly larger than the primary model when adding other impulsive personality traits. Further, increases in other impulsive personality traits at age 14 did not predict increases in internalizing symptoms at age 15, demonstrating specificity of the pathway related to negative urgency at age 14.

**Figure 6.**
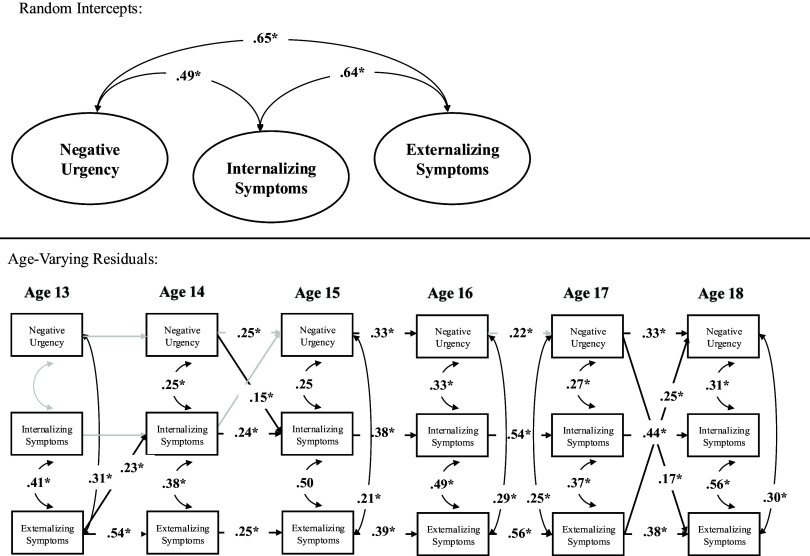
Random intercept cross-lagged panel model including externalizing symptoms. Note. This model a random intercepts of externalizing symptoms, as well as contemporaneous correlated change and prospective relations with negative urgency and internalizing. However, as seen above, primary paths between negative urgency and internalizing were unchanged. There were, however, several pathways between negative urgency and externalzing symptoms worthy of noting. *p < .05.

**Externalizing symptoms.** Sensitivity analyses incorporating externalizing symptoms, which can be seen in Figure 6, fit the data well. Above and beyond random intercept correlations between internalizing and externalizing as well as negative urgency and externalizing, the random intercepts of internalizing symptoms and negative urgency remained statistically significant (see Supplemental Table 3). Correlated change between internalizing symptoms and negative urgency remained across ages 14–18 but not age 13, whereas correlated change between externalizing symptoms and negative urgency was present across age 13–14, and 15–18 but not age 14–15. In terms of prospective pathways, within-person increases in negative urgency at age 14 continued to predict within-person increases in internalizing symptoms at age 15, but negative urgency at age 14 did not to predict within-person increases in externalizing symptoms at age 15. Interestingly, though, there were within-person reciprocal pathways between externalizing symptoms and negative urgency between ages 17–18. However, no significant relations emerged between internalizing symptoms and negative urgency between ages 17–18 (see Figure 6).

### Exploratory analyses

Both parenting (i.e., parental monitoring, involvement) and school transitions (e.g., transitioning from middle school to high school) were theorized to play an important role in study findings. Therefore, exploratory analyses tested the impact of including such variables in models.

**Parenting: Monitoring.** Sensitivity analyses incorporating parental monitoring, which can be seen in supplemental figure 1, fit the data well. Above and beyond random intercept correlations between parental monitoring and lesser negative urgency and internalizing symptoms, the random intercepts of internalizing symptoms and negative urgency remained statistically significant. Correlated change between internalizing symptoms and negative urgency remained across ages 14–18 but not age 13, whereas correlated change between parental monitoring and internalizing symptoms/negative urgency were only present at age 18 (see supplemental material). In terms of prospective pathways, the effect size of within-person increases in negative urgency at age 14 on within-person increases in internalizing symptoms at age 15 was slightly larger than the primary model when including parental monitoring (*b* = .14, SE = .07, *p* = .057), however this effect size had more error and no longer met criteria for statistical significance.

**Parenting: Involvement.** Sensitivity analyses incorporating parental monitoring, which can be seen in Supplemental Figure 2, fit the data well. Above and beyond random intercept correlations between parental involvement and lesser negative urgency as well as parental monitoring and internalizing symptoms, the random intercepts of internalizing symptoms and negative urgency remained statistically significant. Correlated change between internalizing symptoms and negative urgency remained across ages 14–18 but not age 13, whereas correlated change between parenting and internalizing symptoms/negative urgency were sporadic throughout middle adolescence (see supplemental material). In terms of prospective pathways, the effect size of within-person increases in negative urgency at age 14 on within-person increases in internalizing symptoms at age 15 was identical to that of the primary model when including parental involvement (*b* = .13, SE = .08, *p* = .077), however this effect size had more error and no longer met criteria for statistical significance.

**School transitions.** Sensitivity analyses incorporating variability in school transitions, which occurred during ages 13–14, 14–15, and 17–18, fit the data well. The random intercepts of internalizing symptoms and negative urgency remained statistically significant (*b* = .50, SE = .07, *p* < .001). Correlated change between internalizing symptoms and negative urgency remained across ages 14–18 but not age 13. In terms of prospective pathways, the effect size of within-person increases in negative urgency at age 14 on within-person increases in internalizing symptoms at age 15 was identical to that of the primary model when including school transitions (*b* = .13, SE = .08, *p* = .085), however this effect size had more error and no longer met criteria for statistical significance. School transitions at age 17 (i.e., high school to college) predicted within-person decreases in negative urgency at age 18 (*b* = −.11, SE = .05, *p* = .026), however no other relation between school transitions and subsequent negative urgency/internalizing symptoms were statistically significant.

## Discussion

Negative urgency is a transdiagnostic risk factor for a host of mental health concerns (Twenge et al., 2019), yet its developmental etiology and progression remain understudied. Internalizing symptoms are central to theories of negative urgency, in that individuals with higher levels of internalizing symptomatology report higher levels of negative urgency (e.g., Berg et al., 2015), and negative urgency is prospectively associated with internalizing symptoms (e.g., Woods-Gonzalez et al., 2024). However, research has largely treated negative urgency as a stable construct, ignoring any age-related changes in internalizing symptoms and negative urgency or their dynamic relations. These age-varying relations may further elucidate the temporal precedence, and developmental timing, of relations between the two. Therefore, the current study sought to test developmental relations between internalizing symptoms and negative urgency during middle adolescence (age 13–18) via random intercept cross-lagged panel models (RI-CLPMs), effectively parsing stable, “trait-like” variation from time-varying, age-specific variation.

It was hypothesized that the between-person random intercepts of negative urgency and internalizing symptoms would be correlated with one another. This hypothesis was supported, in that the random intercepts between the two were highly correlated. There are several interpretations of these findings. One interpretation is that negative urgency and internalizing were highly correlated due to potential shared genetic features (e.g., Cyders & Smith, 2008; Wang & Chassin, 2018) and thus they manifest as person-level differences from an early age. Another interpretation, however, is that these random intercepts represent substantial confounding between negative urgency and internalizing symptoms, and that other variables explain their between-person co-occurrence. As stated, one such variable may be genetics. However, given that participants were adolescents age 13–18, another such confounding variable may be familial functioning and/or parenting. Research suggests that harsh parenting and lower frequency positive parenting is associated with increased internalizing symptoms (e.g., Dallaire et al., 2006) and negative urgency (e.g., Waddell et al., 2024), and thus between-participants variability in parenting may be one confounding factor. However, it is worth noting that, when including parental monitoring and parental involvement in exploratory models, which measure the opposite of harsh parenting, the random intercept between negative urgency and internalizing symptoms was only slightly changed. While replication is needed, it is likely that other psychosocial factors may rather explain study associations. Another such factor may be stress, in that financial, family, and life stress is associated with both negative urgency and internalizing symptoms across individuals (e.g., Guan et al., 2022; Woods-Gonzalez et al., 2024).

It was also hypothesized that within-person changes in negative urgency and internalizing symptoms would covary with one another. Such hypotheses were supported, in that within-person changes in negative urgency were contemporaneously correlated with within-person changes in internalizing symptoms across all ages except for age 13. The transition into high school is thought to occur around age 14 (e.g., Benner, 2011; Benner et al., 2017). Thus, one interpretation of findings may be that within-person contemporaneous changes in negative urgency and internalizing symptoms occur once adolescents start high school, and such correlated change may be due to underlying shifts in stress, exposure to peer influences, and academic obligations known to increase during the high school years (e.g., Fraser et al., 2021). Rather, levels of negative urgency and internalizing may be more static at age 13 before showing substantial within-person variation thereafter across development. School transitions between ages 13–15 and 17–18 were included as covariates in exploratory models, but correlated change after age 14 was unchanged. However, future research is needed to more deeply explore aspects of these transitions that may shed further light into how (and why) such correlated change was observed during these years.

Interestingly, though, our hypotheses regarding reciprocal longitudinal relations between changes in negative urgency and internalizing symptoms were not supported across the full sample. When specifying models across the full sample of middle adolescents, within-person changes in internalizing symptoms did not predict within-person changes in negative urgency. However, a within-person change in negative urgency at age 14 predicted a within-person change in internalizing symptoms at age 15 but not at any other age. In line with correlated changes observed after age 14, one possibility may be that these within-person changes occurred at-or-around the time of transition into high school (e.g., Benner, 2011; Benner et al., 2017), triggering a substantial uptick in negative urgency which then predicted an increase in internalizing symptoms a year later. This effect was robust in exploratory models that included school transitions as covariates, though, suggesting that the sole transition into a new school environment wasn’t the reason for such relations. While opposing theories suggest that internalizing symptoms predict negative urgency (e.g., Berg et al., 2015; Cyders & Smith, 2008), findings regarding the directional pathway from negative urgency to internalizing symptoms from age 14 to 15 rather speak to a more modern theory of negative urgency wherein urgency may predict subsequent behavioral inaction or avoidance (e.g., Carver & Johnson, 2018), which then naturally predicts upticks in internalizing symptoms (e.g., Fernández-Rodríguez et al., 2018; Venta et al., 2012). That is, since negative urgency is associated with the use of disengagement emotion regulation strategies (e.g., avoiding conflict, avoiding going to an event; King et al., 2018), such strategies may then over time lead to increases in internalizing symptoms given that they reduce immediate internalizing symptoms (e.g., acute anxiety) but rather reinforce increased internalizing symptoms over time. However, future research is needed to more deeply understand why this directional pathway was present from age 14 to 15 but no other ages.

It is important to note that there were strong, consistent autoregressive pathways for both negative urgency and internalizing across ages, except for age 13–14, wherein a within-person change at a given age predicted a subsequent within-person increase one year later, showing a pattern of escalation. Put in the context of findings regarding correlated change, one interpretation may be that a within-person increase in negative urgency correlated with a within-person increase in internalizing symptoms, which then set an individual off onto a cycle of continued increases thereafter well into middle adolescence. However, given that age 14 changes in negative urgency predicted age 15 increases in internalizing symptoms, another interpretation could be that age 14 changes in both negative urgency and internalizing symptoms serve as turning points, during which internalizing symptoms increase the subsequent year and then continue to increase over time. From a prevention standpoint, the present findings suggest that age 14 may be a time of considerable prevention value in attending to internalizing symptoms and negative urgency as they may establish changes in these personal factors for the years to come.

When specifying models within male youth and within female youth findings showed substantial differences. Importantly, the between-person relations between negative urgency and internalizing symptoms were more highly correlated for male youth (*b* = .56) compared to female youth (*b* = .40). However, the prospective within-person pathway from age 14 changes in negative urgency to subsequent age 15 changes in internalizing symptoms was only present in female youth (*b* = .27, *p* = .032) compared to within male youth (*b* = .05, *p* = .55). Further, within female youth, changes in age 13 internalizing symptoms predicted changes in age 14 negative urgency as well. Thus, one interpretation of these findings may be that, within female youth, increased internalizing symptoms at age 13 predict increased negative urgency at age 14, which then predicts increased internalizing symptoms at age 15 and beyond, creating a cycle toward persistent increases in internalizing symptoms thereafter. Further, while negative urgency and internalizing symptoms may play a stronger age-varying role in predicting subsequent increases in one another for female youth from age 13 to 15, it appears the two co-exist across middle adolescence to a stronger degree for male youth. One possibility for such findings may be that male youth’s levels of negative urgency and internalizing symptoms are more stable over time, whereas female youth’s levels fluctuate to a higher degree. In support, research suggests that female youth have more pronounced dynamic changes in internalizing symptoms across middle adolescence compared to boys (Fernandez Castelao & Kröner-Herwig, 2013). Thus, when significant fluctuations in negative urgency do occur at age 14, across the sample, fluctuations likely have a large impact on further within-person changes in female youth as compared to male youth. Rather, at the between-person level, one explanation may be that male youth possess traits that may increase the link between negative urgency and internalizing symptoms, such as less adaptive coping strategies (e.g., Eschenbeck et al., 2007).

Sensitivity analyses largely replicated the primary model in individuals who showed elevated internalizing symptoms throughout middle adolescence. Of note, the effect size of age 14 increases in negative urgency on age 15 increases in internalizing was augmented in this group, albeit this effect size had more error surrounding it and was not statistically significant. While this subsample likely had limited power, with *N* = 351 individuals showing elevated internalizing symptoms, such analyses demonstrated preliminary evidence that this effect size may be most pronounced in those with elevated symptoms. Sensitivity analyses incorporating other impulsive personality traits showed specificity of findings, given that all relations between negative urgency and internalizing remained when incorporating other impulsive personality traits. Of particular note, changes in other impulsive personality traits at age 14 did not predict subsequent age 15 increases in internalizing symptoms, and the correlations among the random intercept of negative urgency and internalizing symptoms was the largest and most consistent across all impulsive personality traits. Thus, while relations between impulsivity more broadly and internalizing symptoms have been established (Cosi et al., 2011; Johnson et al., 2017), such analyses demonstrated that the paths found in the current study were specific to negative urgency.

Finally, sensitivity analyses incorporating externalizing symptoms showed that none of the observed pathways between internalizing symptoms and negative urgency dissipated when accounting for externalizing symptoms. This is particularly noteworthy, given the large correlations present in the current study, both across people and ages, between internalizing and externalizing symptoms. While not the basis of the current study, several interesting pathways specific to externalizing were demonstrated. Across people, the random intercepts of externalizing symptoms and negative urgency were highly correlated, and there were reciprocal relations between age 17 increases in negative urgency/externalizing symptoms and subsequent age 18 deviations in the externalizing symptoms/negative urgency. One interpretation of such findings may be that prospective, age-varying relations between negative urgency and internalizing symptoms occur earlier during middle adolescence (ages 14–15) during likely school transitions (e.g., Lippold et al., 2013), whereas prospective age-varying relations between negative urgency and externalizing symptoms occur later during middle adolescence (ages 17–18) during likely transitions into riskier behavior (e.g., Oman et al., 2002).

Findings may have implications for theoretical models and personalized early interventions. In terms of theory, findings suggest that there is substantial shared variability between negative urgency and internalizing symptoms, and changes in the two co-vary. However, directional effects were specific to female youth, when age 13 increases in internalizing symptoms predict increases in age 14 negative urgency, and increases in age 14 negative urgency predict increases in age 15 internalizing symptoms. Thus, findings do not necessarily support the notion that internalizing symptoms precede negative urgency across the sample (e.g., Berg et al., 2015), but rather only in female youth when there were reciprocal relations between ages 13–15. In terms of prevention, findings may suggest that targeted interventions for individuals who have higher levels of internalizing and/or negative urgency may have additional benefits on the other, given strong between-person correlations. However, findings also indicate that monitoring within-person changes in internalizing symptoms and negative urgency around ages 13–14 and coupling such changes with adaptive interventions, such as coping-focused skills (e.g., Zapolski et al., 2021; Zapolski & Smith, 2017), may interrupt the link with subsequent changes in both negative urgency and internalizing symptoms. Further, research suggests that personality-focused interventions, such as those that provide feedback based upon an individual’s self-reported scores on personality traits and tailored interventions for certain personality profiles (e.g., PreVenture; Edalati & Conrod, 2019; Lawler et al., 2024), show efficacy in reducing internalizing symptoms (Newton et al., 2020). Thus, such interventions may benefit from additional specificity in feedback and personality-centered content related to negative urgency.

Study findings must be interpreted alongside limitations. Although the current study provided age-specific information regarding negative urgency and internalizing symptoms, using annual assessments may ignore their dynamic fluctuations and time-sensitive effects of youth behavior on a shorter timescale. While this study design allowed for a proper view of developmental changes over a six-year period across ages 13–18, there may be micro-level changes happening within a given assessment year that are undetectable by such yearly assessments. Future studies may consider incorporating temporally nuanced assessment schedules, such as monthly surveys and/or ecological momentary assessments (EMA). Similarly, while the current design provided a snapshot of data during a critical period of adolescent development (i.e., ages 13–18), it did not encompass the full development of negative urgency or internalizing symptoms that may occur well into young adulthood and beyond. Young adulthood is a time of further neurobiological, emotional, and social changes, which are often characterized by identity exploration and increased agency (e.g., Wood et al., 2017), and thus future research is needed to test reciprocal relations during young adulthood as well.

Another limitation is the current sample’s generalizability to demographics not studied herein. In the current study, a majority of participants identified as White (71%). While the findings offer valuable insight into adolescent development, the limited sociodemographic variability of the sample may limit the generalizability of these results to more diverse populations. Finally, it is important to consider the role that educational settings may have played in shaping adolescent development of negative urgency and internalizing symptoms. For example, educational and schooling may have had important, yet complicated, effects on the developmental patterns observed in this study. While we ruled out the timing of school transitions as an explanatory factor, whether a participant is living with their parents versus other caregivers, whether a participant is a high academic achiever, and/or whether a participant faces bullying and/or social acceptance at school, for example, may have considerable impact. Thus, future research is needed to understand the impact of schooling/education on study findings. Finally, the current study used the RI-CLPM, which represents a significant advance in our ability to test within- and between-person longitudinal associations (Hamaker et al., 2015). However, one downfall of the RI-CLPM is that it is quite sensitive to sample size issues, and requires large samples for adequate power (e.g., Hamaker et al., 2015; Hamaker, 2023). Thus, future research in additional samples is needed to replicate study findings.

Despite study limitations, the current study advances theory and etiological models of negative urgency, internalizing symptoms, and their co-occurrence. Findings suggest that negative urgency and internalizing symptoms co-occur across individuals during middle adolescence, and that they change concurrently within an individual. However, changes in negative urgency at age 14 had a prospective effect on changes in internalizing symptoms occurring at age 15, suggesting that age 14 is a particularly important time to understand and target changes in negative urgency. Future research using more temporal assessments of negative urgency and internalizing symptoms that occur shortly before or after transitions in adolescent and young adult development (e.g., into and out of high school, into and out of college) is needed.

## Supporting information

10.1017/S0954579426101357.sm001 Waddell et al. supplementary material Waddell et al. supplementary material

## Data Availability

Access to NCANDA data is available via the National Institute on Alcohol Abuse and Alcoholism (NIAAA) Data Archive (collection 4513, https://nda.nih.gov/edit_collection.html?id=4513). Study analyses were not pre-registered.

## Supplementary material

The supplementary material for this article can be found at https://doi.org/10.1017/S0954579426101357.

## Pre-registration statement

Study analyses were not pre-registered due to oversight at the study planning stage.
