# Supplementary material for: Developmental relations between internalizing symptoms and negative urgency during middle adolescence
Source: Dev Psychopathol. 2026 Mar 27:1–16. Online ahead of print. doi: 10.1017/S0954579426101357 (PMC13038404; doi:10.1017/S0954579426101357)
Supplement: Waddell et al. supplementary material [file S0954579426101357sup001.docx]

| **Supplemental Table 1:** RI-CLPM Parameters For Clinical Level Internalizing Symptoms | | | |
| --- | --- | --- | --- |
|  | b | SE | p-value |
| **Random Intercepts** |  |  |  |
| Negative Urgency – Internalizing | .51 | .13 | <.001 |
| **Predictors of Random Intercepts** |  |  |  |
| Negative Urgency |  |  |  |
| Socioeconomic Status | -.10 | 09 | .26 |
| Sex | .01 | .08 | .98 |
| Internalizing Symptoms |  |  |  |
| Socioeconomic Status | -.11 | .09 | .23 |
| Sex | -.21 | .10 | .04 |
| **Correlated Change** |  |  |  |
| Age 13 Negative Urgency – Internalizing | .21 | .15 | .15 |
| Age 14 Negative Urgency – Internalizing | .27 | .14 | .048 |
| Age 15 Negative Urgency – Internalizing | .42 | .11 | <.001 |
| Age 16 Negative Urgency – Internalizing | .43 | .07 | <.001 |
| Age 17 Negative Urgency – Internalizing | .28 | .09 | .002 |
| Age 18 Negative Urgency – Internalizing | .56 | .09 | <.001 |
| **Autoregressive Pathways** |  |  |  |
| Age 13 Negative Urgency 🡪 Age 14 Negative Urgency | .18 | .17 | .31 |
| Age 14 Negative Urgency 🡪 Age 15 Negative Urgency | .15 | .15 | .31 |
| Age 15 Negative Urgency 🡪 Age 16 Negative Urgency | .27 | .12 | .025 |
| Age 16 Negative Urgency 🡪 Age 17 Negative Urgency | .20 | .12 | .098 |
| Age 17 Negative Urgency 🡪 Age 18 Negative Urgency | .47 | .12 | <.001 |
| Age 13 Internalizing 🡪 Age 14 Internalizing | .30 | .15 | .041 |
| Age 14 Internalizing 🡪 Age 15 Internalizing | .32. | .12 | .009 |
| Age 15 Internalizing 🡪 Age 16 Internalizing | .42. | .12 | <.001 |
| Age 16 Internalizing 🡪 Age 17 Internalizing | .52 | .09 | <.001 |
| Age 17 Internalizing 🡪 Age 18 Internalizing | .36 | .13 | .006 |
| **Prospective Pathways** |  |  |  |
| Age 13 Negative Urgency 🡪 Age 14 Internalizing | -.03 | .16 | .84 |
| Age 14 Negative Urgency 🡪 Age 15 Internalizing | .19 | .10 | .056 |
| Age 15 Negative Urgency 🡪 Age 16 Internalizing | -.01 | .12 | .92 |
| Age 16 Negative Urgency 🡪 Age 17 Internalizing | .13 | .12 | .26 |
| Age 17 Negative Urgency 🡪 Age 18 Internalizing | .11 | .12 | .34 |
| Age 13 Internalizing 🡪 Age 14 Negative Urgency | .12 | .18 | .49 |
| Age 14 Internalizing 🡪 Age 15 Negative Urgency | .23 | .12 | .061 |
| Age 15 Internalizing 🡪 Age 16 Negative Urgency | .11 | .12 | .37 |
| Age 16 Internalizing 🡪 Age 17 Negative Urgency | .13. | .11 | 24 |
| Age 17 Internalizing 🡪 Age 18 Negative Urgency | -.17 | .16 | .28 |
| *Note.* RI-CLPM stands for random intercept cross-lagged panel model. | | | |

| **Supplemental Table 2:** RI-CLPM Parameters When Covarying Other UPPS-P Traits | | | |
| --- | --- | --- | --- |
|  | b | SE | p-value |
| **Random Intercepts** |  |  |  |
| Negative Urgency – Internalizing | .52 | .08 | <.001 |
| **Predictors of Random Intercepts** |  |  |  |
| Negative Urgency |  |  |  |
| Socioeconomic Status | -.16 | .05 | .003 |
| Sex | .04 | .05 | .39 |
| Internalizing Symptoms |  |  |  |
| Socioeconomic Status | -.09 | .05 | .075 |
| Sex | -.11 | .05 | .03 |
| **Correlated Change** |  |  |  |
| Age 13 Negative Urgency – Internalizing | .17 | .13 | .20 |
| Age 14 Negative Urgency – Internalizing | .23 | .10 | .02 |
| Age 15 Negative Urgency – Internalizing | .24 | .08 | .003 |
| Age 16 Negative Urgency – Internalizing | .30 | .06 | <.001 |
| Age 17 Negative Urgency – Internalizing | .26 | .05 | <.001 |
| Age 18 Negative Urgency – Internalizing | .31 | .07 | <.001 |
| **Autoregressive Pathways** |  |  |  |
| Age 13 Negative Urgency 🡪 Age 14 Negative Urgency | -.09 | .14 | .51 |
| Age 14 Negative Urgency 🡪 Age 15 Negative Urgency | .20 | .12 | .097 |
| Age 15 Negative Urgency 🡪 Age 16 Negative Urgency | .30 | .09 | .001 |
| Age 16 Negative Urgency 🡪 Age 17 Negative Urgency | .19 | .08 | .013 |
| Age 17 Negative Urgency 🡪 Age 18 Negative Urgency | .30 | .08 | <.001 |
| Age 13 Internalizing 🡪 Age 14 Internalizing | .19 | .13 | .16 |
| Age 14 Internalizing 🡪 Age 15 Internalizing | .19 | .10 | .063 |
| Age 15 Internalizing 🡪 Age 16 Internalizing | .32 | .11 | .003 |
| Age 16 Internalizing 🡪 Age 17 Internalizing | .42 | .11 | <.001 |
| Age 17 Internalizing 🡪 Age 18 Internalizing | .40 | .10 | <.001 |
| **Prospective Pathways** |  |  |  |
| Age 13 Negative Urgency 🡪 Age 14 Internalizing | -.07 | .16 | .64 |
| Age 14 Negative Urgency 🡪 Age 15 Internalizing | .17 | .08 | .04 |
| Age 15 Negative Urgency 🡪 Age 16 Internalizing | .06 | .09 | .50 |
| Age 16 Negative Urgency 🡪 Age 17 Internalizing | .06 | .08 | .41 |
| Age 17 Negative Urgency 🡪 Age 18 Internalizing | .04 | .07 | .55 |
| Age 13 Internalizing 🡪 Age 14 Negative Urgency | .19 | .13 | .16 |
| Age 14 Internalizing 🡪 Age 15 Negative Urgency | -.10 | .09 | .28 |
| Age 15 Internalizing 🡪 Age 16 Negative Urgency | .09 | .07 | .24 |
| Age 16 Internalizing 🡪 Age 17 Negative Urgency | .07 | .08 | .35 |
| Age 17 Internalizing 🡪 Age 18 Negative Urgency | .08 | .09 | .38 |
| *Note.* RI-CLPM stands for random intercept cross-lagged panel model. | | | |

| **Supplemental Table 3:** RI-CLPM Parameters When Covarying Other UPPS-P Traits | | | |
| --- | --- | --- | --- |
|  | b | SE | p-value |
| **Random Intercepts** |  |  |  |
| Negative Urgency – Internalizing | .49 | .08 | <.001 |
| Negative Urgency – Externalizing | .65 | .06 | <.001 |
| Externalizing – Internalizing | .64 | .07 | <.001 |
| **Predictors of Random Intercepts** |  |  |  |
| Negative Urgency |  |  |  |
| Socioeconomic Status | -.16 | .05 | .003 |
| Sex | .04 | .05 | .45 |
| Internalizing Symptoms |  |  |  |
| Socioeconomic Status | -.10 | .05 | .056 |
| Sex | -.13. | 05 | .012 |
| Externalizing Symptoms |  |  |  |
| Socioeconomic Status | -.13 | .05 | .016 |
| Sex | -.03 | .05 | .54 |
| **Correlated Change** |  |  |  |
| Age 13 Negative Urgency – Internalizing | .14 | .12 | .24 |
| Age 14 Negative Urgency – Internalizing | .25 | .09 | .005 |
| Age 15 Negative Urgency – Internalizing | .25 | .08 | .001 |
| Age 16 Negative Urgency – Internalizing | .33 | .05 | <.001 |
| Age 17 Negative Urgency – Internalizing | .27 | .06 | <.001 |
| Age 18 Negative Urgency – Internalizing | .31 | .06 | <.001 |
| Age 13 Negative Urgency – Externalizing | .31 | .11 | .007 |
| Age 14 Negative Urgency – Externalizing | .13 | .10 | .21 |
| Age 15 Negative Urgency – Externalizing | .21 | .08 | .008 |
| Age 16 Negative Urgency – Externalizing | .29 | .06 | <.001 |
| Age 17 Negative Urgency – Externalizing | .25 | .07 | <.001 |
| Age 18 Negative Urgency – Externalizing | .30 | .05 | <.001 |
| Age 13 Externalizing – Internalizing | .41 | .10 | <.001 |
| Age 14 Externalizing – Internalizing | .38 | .10 | <.001 |
| Age 15 Externalizing – Internalizing | .50 | .07 | <.001 |
| Age 16 Externalizing – Internalizing | .49 | .05 | <.001 |
| Age 17 Externalizing – Internalizing | .37 | .06 | <.001 |
| Age 18 Externalizing – Internalizing | .56 | .04 | <.001 |
| **Autoregressive Pathways** |  |  |  |
| Age 13 Negative Urgency 🡪 Age 14 Negative Urgency | -.03 | .13 | .85 |
| Age 14 Negative Urgency 🡪 Age 15 Negative Urgency | .25 | .10 | .01 |
| Age 15 Negative Urgency 🡪 Age 16 Negative Urgency | .33 | .08 | <.001 |
| Age 16 Negative Urgency 🡪 Age 17 Negative Urgency | .22 | .08 | .003 |
| Age 17 Negative Urgency 🡪 Age 18 Negative Urgency | .33 | .07 | <.001 |
| Age 13 Internalizing 🡪 Age 14 Internalizing | .16 | .12 | .20 |
| Age 14 Internalizing 🡪 Age 15 Internalizing | .24 | .11 | .022 |
| Age 15 Internalizing 🡪 Age 16 Internalizing | .38 | .10 | <.001 |
| Age 16 Internalizing 🡪 Age 17 Internalizing | .54 | .08 | <.001 |
| Age 17 Internalizing 🡪 Age 18 Internalizing | .44 | .07 | <.001 |
| Age 13 Externalizing 🡪 Age 14 Externalizing | .54 | .12 | <.001 |
| Age 14 Externalizing 🡪 Age 15 Externalizing | .25 | .12 | .03 |
| Age 15 Externalizing 🡪 Age 16 Externalizing | .39 | .12 | .001 |
| Age 16 Externalizing 🡪 Age 17 Externalizing | .56 | .09 | <.001 |
| Age 17 Externalizing 🡪 Age 18 Externalizing | .38 | .09 | <.001 |
| **Prospective Pathways** |  |  |  |
| Age 13 Negative Urgency 🡪 Age 14 Internalizing | -.13 | .11 | .23 |
| Age 14 Negative Urgency 🡪 Age 15 Internalizing | .15 | .07 | .041 |
| Age 15 Negative Urgency 🡪 Age 16 Internalizing | -.01 | .08 | .88 |
| Age 16 Negative Urgency 🡪 Age 17 Internalizing | .07 | .07 | .30 |
| Age 17 Negative Urgency 🡪 Age 18 Internalizing | .01 | .06 | .96 |
| Age 13 Negative Urgency 🡪 Age 14 Externalizing | -.21 | .12 | .09 |
| Age 14 Negative Urgency 🡪 Age 15 Externalizing | .10 | .10 | .30 |
| Age 15 Negative Urgency 🡪 Age 16 Externalizing | -.01 | .08 | .92 |
| Age 16 Negative Urgency 🡪 Age 17 Externalizing | .11 | .07 | .088 |
| Age 17 Negative Urgency 🡪 Age 18 Externalizing | .17 | .07 | .009 |
| Age 13 Internalizing 🡪 Age 14 Negative Urgency | .10 | .13 | .45 |
| Age 14 Internalizing 🡪 Age 15 Negative Urgency | -.09 | .09 | .35 |
| Age 15 Internalizing 🡪 Age 16 Negative Urgency | .06 | .09 | .52 |
| Age 16 Internalizing 🡪 Age 17 Negative Urgency | .04 | .08 | .65 |
| Age 17 Internalizing 🡪 Age 18 Negative Urgency | .04 | .07 | .54 |
| Age 13 Internalizing 🡪 Age 14 Externalizing | -.04 | .12 | .75 |
| Age 14 Internalizing 🡪 Age 15 Externalizing | -.01 | .12 | .99 |
| Age 15 Internalizing 🡪 Age 16 Externalizing | -.05 | .10 | .64 |
| Age 16 Internalizing 🡪 Age 17 Externalizing | -.12 | .08 | .13 |
| Age 17 Internalizing 🡪 Age 18 Externalizing | .02 | .08 | .78 |
| Age 13 Externalizing 🡪 Age 14 Negative Urgency | .17 | .11 | .13 |
| Age 14 Externalizing 🡪 Age 15 Negative Urgency | .10 | .10 | .30 |
| Age 15 Externalizing 🡪 Age 16 Negative Urgency | .10 | .09 | .25 |
| Age 16 Externalizing 🡪 Age 17 Negative Urgency | .09 | .08 | .25 |
| Age 17 Externalizing 🡪 Age 18 Negative Urgency | .25 | .07 | .001 |
| Age 13 Externalizing 🡪 Age 14 Internalizing | .23 | .11 | .04 |
| Age 14 Externalizing 🡪 Age 15 Internalizing | -.06 | .10 | .52 |
| Age 15 Externalizing 🡪 Age 16 Internalizing | -.03 | .11 | .80 |
| Age 16 Externalizing 🡪 Age 17 Internalizing | -.11 | .08 | .17 |
| Age 17 Externalizing 🡪 Age 18 Internalizing | .09 | .07 | .22 |
| *Note.* RI-CLPM stands for random intercept cross-lagged panel model. | | | |

**Supplemental Figure 1:** Random Intercept Cross-Lagged Panel Model Including Parental Monitoring

*Note.* This model has random intercepts of parental monitoring, as well as contemporaneous correlated change and prospective relations with negative urgency and internalizing. However, as seen above, primary paths between negative urgency and internalizing were largely unchanged. * p < .05

**Supplemental Figure 2:** Random Intercept Cross-Lagged Panel Model Including Parental Involvement

*Note.* This model has random intercepts of parental invovlement, as well as contemporaneous correlated change and prospective relations with negative urgency and internalizing. However, as seen above, primary paths between negative urgency and internalizing were largely unchanged. * p < .05
